# Tissue-specific gene expression templates for accurate molecular characterization of the normal physiological states of multiple human tissues with implication in development and cancer studies

**DOI:** 10.1186/1471-2164-12-439

**Published:** 2011-09-01

**Authors:** Pei-Ing Hwang, Huan-Bin Wu, Chin-Di Wang, Bai-Ling Lin, Cheng-Tao Chen, Shinsheng Yuan, Guani Wu, Ker-Chau Li

**Affiliations:** 1 Institute of Statistical Science, Academia Sinica, Taipei, Taiwan 115, Republic of China; 2 Genome Research Center, Academia Sinica, Taipei, Taiwan 115, Republic of China; 3 Department of Statistics, University of California, Los Angeles, Los Angeles, California 90095, USA

## Abstract

**Background:**

To elucidate the molecular complications in many complex diseases, we argue for the priority to construct a model representing the normal physiological state of a cell/tissue.

**Results:**

By analyzing three independent microarray datasets on normal human tissues, we established a quantitative molecular model GET, which consists of 24 tissue-specific *G*ene *E*xpression *T*emplates constructed from a set of 56 genes, for predicting 24 distinct tissue types under disease-free condition. 99.2% correctness was reached when a large-scale validation was performed on 61 new datasets to test the tissue-prediction power of GET. Network analysis based on molecular interactions suggests a potential role of these 56 genes in tissue differentiation and carcinogenesis.

Applying GET to transcriptomic datasets produced from tissue development studies the results correlated well with developmental stages. Cancerous tissues and cell lines yielded significantly lower correlation with GET than the normal tissues. GET distinguished melanoma from normal skin tissue or benign skin tumor with 96% sensitivity and 89% specificity.

**Conclusions:**

These results strongly suggest that a normal tissue or cell may uphold its normal functioning and morphology by maintaining specific chemical stoichiometry among genes. The state of stoichiometry can be depicted by a compact set of representative genes such as the 56 genes obtained here. A significant deviation from normal stoichiometry may result in malfunction or abnormal growth of the cells.

## Background

It has been well-recognized that within a cell, not only genes participate in cascades of biochemical events (pathways), but also the pathways themselves cross-talk with each other as a delicate and intriguing network system. Such complexity was reflected in the normal biological processes (tissue development, for example) as well as in the complex disease processes such as autism, cancer, rheumatoid arthritis and coronary artery disease [1,2]. In addition, the genetic interactions of oncogenes and tumor suppressor genes may perturb the normal network system through a variety of altered molecular properties of the normal genes, magnifying the difficulties encountered in the cancer biology study [3]. Because of this, it is important to develop quantitative molecular models which can represent different physiological or pathological states of a complex biological system and can be used to predict the related states, using high throughput molecular data. In line with this viewpoint, we argue for the priority to construct models for the normal physiological states first. This is because (1) normal cells/tissues are endowed with the most stable biochemical homeostasis and (2) such models may serve as general references for contrasting with various pathological or altered physiological states.

Up to now, in part due to the limitation in sample availability, few studies on normal human tissues have been reported. Through the transcriptome study of the disease-free human samples via microarray analysis, gross patterns of tissue-gene relationships have been observed by several teams [4-7]. A recent study which applied statistical and network analysis to transcriptomic data from 31 normal human tissue types has resulted in putative tissue-specific networks for nine tissues. These putative tissue-specific networks were suggested as potential drug targets [8]. However, it still awaits a deeper investigation to find out what molecular signatures can best represent the normal state of a specific tissue and offer the most transparent and systematic elucidation on tissue differences (regarding anatomy, pathology and development). In this study, by re-analyzing some of the transcriptomic datasets produced from normal human tissues in the Gene Expression Omnibus (GEO), we identified a set of 56 genes whose transcript profiles are endowed with strong tissue-specific properties for 24 different tissue types under the disease-free condition. These genes present significant variation of expression amongst tissues. From the expression level of these 56 genes, we constructed 24 tissue-specific *G*ene *E*xpression *T*emplates (GETs), one for each of the 24 tissues. We first validated that these GETs can differentiate tissue types under the normal physiological condition. Then we demonstrated how GET can be applied under other conditions, including development and cancerous conditions. Our results suggest that homeostasis among various molecules in a cell/tissue may play a key role in maintaining its normal functioning and the homeostasis state can be characterized by the 56 genes.

## Results

### Characterization of 24 tissue types by the 56 genes

We searched for a set of genes whose expression profile could best represent normal state of a specific tissue type. We used three large-scale microarray datasets as our training datasets to identify a group of 56 genes with high variation in expression across different tissue types (Additional file 1: Table S1). Briefly, we selected the probe sets with coefficient of variation (CV) ranked within the top 2.5% of the entire transcriptome across all samples from each of the three training datasets. After intersecting the three groups of highly variably expressed probe sets, we removed redundant probe sets that share similar expression patterns. Our procedure yielded a set of 56 genes. [see methods for more details].

**Figure 1:**
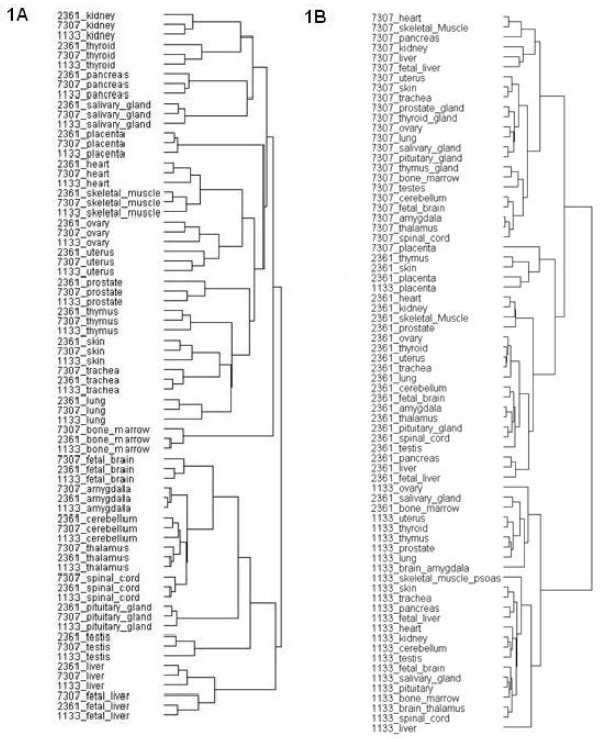
**Dendrograms showing tissue classification by hierarchical clustering analysis with the 56 genes**. The dendrograms shown were generated with standard two-way hierarchical clustering analysis of GenePattern using the expression values of the (A) 56 signature genes or (B) randomly selected 56 genes extracted from the 24 tissues under the three GEO datasets. The associated heatmaps are displayed in Additional file 1: Fig. S1 and Fig. S2, respectively.

Function enrichment analysis, based on an ontology search against the Panther database, showed that these 56 genes were most enriched in encoding cytoskeletal proteins, calmodulin-related proteins, and neuropeptides (*P *value < 1 × 10^-5^). Gene Ontology search indicated that the proteins encoded by the 56 genes were mostly localized to extracellular regions (21/56, *P *value < 3.1 × 10^-5^) and were involved in the processes of response to wounding (10/56, *P *value = 6.12 × 10^-5^), response to steroid hormone stimulus (6/56, *P *value = 4.67 × 10^-4^) and regulation of homeostatic process (5/56, *P *value = 5.94 × 10^-4^). While searching KEGG for the pathways mapped by the 56 genes, we found focal adhesion, ECM-receptor interaction, and PPAR signaling pathway over-represented. (Additional file 1: Table S2) We conducted hierarchical clustering with the 56 genes from the 24 tissue types shared by our three training datasets. The result showed that all of the 24 tissues were well grouped (i.e. the same tissue from three different data sources were grouped together as a cluster; see the dendrogram in Figure 1A and the heat map in Additional file 1: Fig. S1). To confirm that the tissue-clustering result is specific to the gene set we identified, we randomly-selected 56 probe sets and applied the same hierarchical clustering analysis to their expression profiles. We were no longer able to find the tissue clusters; instead, the only visible clusters were three large classes corresponding to tissues from each data source. (Figure 1B; also see Additional file 1: Fig. S2 for heat map) In addition, as suggested by one anonymous reviewer, we also selected the probes with the highest expression level across all tissues as a contrast group. This contrast group of genes (including many house-keeping genes) failed to group tissues properly. (see Additional file 1: Fig. S3)

Taking a closer look at the outcome of the tissue classification obtained from the hierarchical clustering analysis, similar to the previous reports published by Shyamsundar *et al *[6] and Ge *et al *[3] on tissue classification, our results also showed that the tissues were grouped largely by their physiological functions, anatomical locations or cellular composition. For example, ovary and uterus, the organs from the female productive system, were clustered together; the gland tissues/organs including thyroid, pancreas, and salivary gland also aggregated and the testis as previously reported [4,7,9] was grouped with the tissues from the central nervous system like cerebellum, amygdala, and thalamus. However, it should be noted that we obtained the similar tissue classification result with far smaller set of genes (56) as the classifiers, in comparison with the two previous reports where they used 7396 [3] genes and 5592 [6] genes on 36 tissue types (from 36 samples) and 45 tissue types (from 115 specimens), respectively.

### Accurate prediction of normal tissues

We speculated that the expression profile of this compact 56-gene set might represent a set of important molecular features for tissue specification under normal conditions. To determine whether this gene set could accurately predict the tissue type of a sample of unknown origin, the expression profiles of the 56 genes from the 24 tissues were extracted from our three microarray datasets to form the tissue-specific gene expression templates (GET) for tissue prediction. More precisely, for each of the 24 tissues, we obtained a GET which consisted of the average of the probe values of the three datasets for the 56 selected genes. To predict the tissue type of a new sample, we compared the expression pattern of the 56 genes in the new sample with each of the 24 GETs by calculating their correlation coefficients (c.f.). The tissue type of the GET showing the highest degree of similarity as measured by c.f. is our prediction. As an example, we took a new dataset from Gene Expression Omnibus, GSE5364[10], which contained data derived from human liver, lung, and thyroid, in non-malignant and malignant conditions. Tissue prediction was performed by calculating the correlation between the expressions of the 56 genes in each test tissue against each of our 24 GETs. For the non-malignant tissues, we obtained 100% correct prediction, strongly endorsing our speculation that the expression profiles of the 56 genes may be used as molecular signatures for normal human tissues. (Additional file 1: Table S3) To further validate the use of these tissue-specific GETs as predictors of normal human tissues, an extensive tissue-prediction analysis was carried out on a total of 61 microarray datasets consisting of 797 samples from 16 different human tissues. These datasets were obtained by searching the entire GEO database using two criteria: (1) the tissue types must fall into the category of the claimed 24 distinguishable normal human tissues (2) they were hybridized on one of two Affymetrix GeneChips, HG-U133A (GPL96) or HG-U133plus2.0 (GPL570). Cell lines or specific cell types from an organ were excluded (see Methods for details). As it turned out, the overall prediction accuracy of our method already reached 99.2% (791/797). (Table 1 and Additional file 1: Table S4). This large-scale validation demonstrated that the tissue-specific profiles of the 56 genes are essential for the 24 human tissue types. It also suggests that the tissue-specific GETs very likely represent the overall biochemical equilibrium reached by all the molecules of a tissue under normal physiological condition.

**Table 1:** Large-scale prediction of normal human tissues with GETs

Tissue	Datasets	Samples	Correctly predicted	% Accuracy
bone marrow	1	11	11	100%

fetal liver	1	6	6	100%

heart	3	28	28	100%

kidney	11	108	106	99%

liver	9	84	84	100%

lung	11	170	169	99%

ovary	1	4	4	100%

pancrease	2	17	14	82%

pituitary gland	1	1	1	100%

placenta	4	34	34	100%

prostate	3	10	10	100%

skeletal muscle	8	134	134	100%

skin	10	121	121	100%

testis	1	6	6	100%

thyroid	4	36	36	100%

Uterus	1	27	27	100%

Total	61	797	791	99.25%

Samples: number of tested samples derived from normal human tissues.

Correctly Predicted: the number of tested samples for which the tissue source was correctly predicted. The source data as well as the analysis results from each dataset can be found at the Additional file 1: Table S4.

To show how stable and robust the relative expression level of the 56 genes is, we used Spearman's rank correlation to replace the standard Pearson correlation in the tissue-prediction analysis and obtained 96.2% (767/797) of accuracy (Additional file 1: Table S5). It indicates that the relative expression of the 56 genes is a robust feature for characterizing the normal state of a specific human tissue.

So far, we have chosen Affymetrix as the gene expression platform to test the performance of GET. Our reasons are (1) Affymetrix is the common platform used in our three training datasets which ensures the inclusion of the entire set of our 56 genes; (2) the data preprocessing procedure is standardized; (3) existence of many datasets in the public domain for validation and (4) high reproducibility. However, it remains questionable whether GET can be applied to other platforms of measuring gene expression or not. To address this issue, we searched GEO for datasets containing enough samples from norm human tissues. We found an ABI array generated dataset, GSE7905 [8], which contains many normal human tissue samples. Among them, there are 60 samples (20 tissue types in triplicates) matching our 24 tissue types. We treated the data generated by these ABI array as if they were from the Affymetrix and applied GET to make tissue prediction. Strikingly, we found that our Affymetrix-based GETs yield a perfect result, 100% (60/60) (Additional file 1: Table S6). This demonstrates that GET is platform-independent.

### Network analysis reveals involvement of the 56 genes in development and tumorigenesis

**Figure 2:**
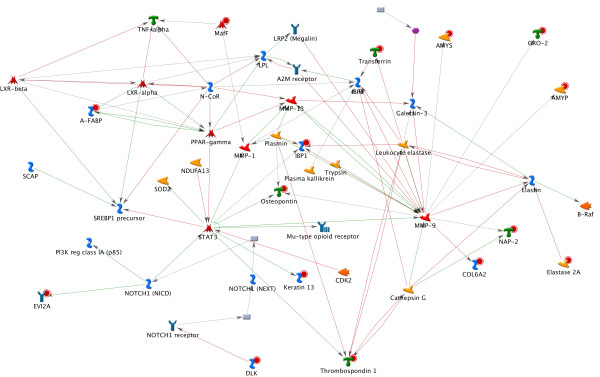
**Network analysis on the 56 genes using MetaCore**. This network is the highest scored one when constructed on the basic algorithm "analyze networks" of MetaCore from GeneGo Inc. using the 56 genes as input. The "analyze networks" algorithm of MetaCore builds on the Dijkstra's algorithm which takes a list of root nodes to create shortest paths networks for each of the nodes to others. The network is stopped while reaching a predefined size [11].

**Figure 3:**
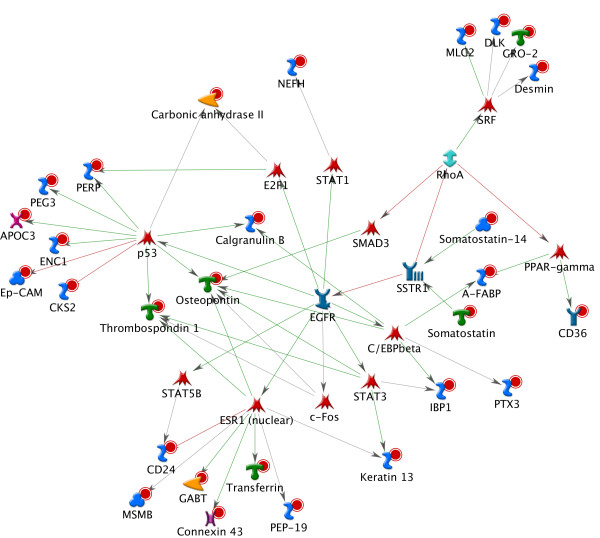
**Network analysis with the 56 genes and the mostly linked transcription factors**. The gene network was built based on the algorithm "Receptor Targets Modeling" which identifies the important transcription factors (TFs) connected to the query genes and the signaling receptors associated with these TFs. Briefly, the algorithm at first builds two lists with the TFs or receptors which have direct links to the input objects and then draws shortest paths from each of the found receptors to each TF. Finally, a series of networks were generated as one network per receptor. Shown here is the highest ranked result. It suggests potential roles of the 56 genes in tissue development and tumorigenesis.

The above results indicate that these tissue-specific GETs may be more than simply a set of biomarkers capable of distinguishing different tissue types. Their profiles may represent a "net sum" of the interplays among the complex gene regulation pathways occurring in a tissue. We explored the possible biological roles of the 56 genes by performing network analysis using the commercial tool MetaCore from GeneGo Inc. which builds gene networks based on molecular interactions acquired from experiments-based literature reports [11]. We firstly applied the basic algorithm "analyze networks" to our 56 genes using the default parameter settings. The top scored results (Figure 2) showed MMP9 (matrix metallopeptidase 9), STAT3 (signal transducer and activator of transcription 3), and PPARG (peroxisome proliferator-activated receptor gamma) to be the most connected molecules in the network (z Score = 65) with 15 of our genes included. MMP9 has been known to be the key regulator for bone remodeling [12,13], STAT3 is involved in embryogenesis, hematopoietic cell development and is a biomarker for embryo stem cells[14-16], and PPARG is involved in the regulation of neural stem cells proliferation and differentiation [17,18]. We then applied the algorithm "Receptor Targets Modeling" which allows users to identify the important transcription factors (TFs) connected to the query genes and the signaling receptors associated with these TFs". The best result produced by this algorithm delivered a network (Z score = 131) (Figure 3) with half (28) of our genes connected to the TFs: STAT3, ESR, SRF, CEBPB, E2F1, PPARG and TP53 and these transcription factors were regulated by EGFR. All of these molecules are known to be important in development and/or tumorigenesis. For example, ESR (estrogen receptor), a ligand-activated transcription factor, is essential for sexual development and reproductive function, and is involved in breast cancer, endometrial cancer, and osteoporosis. The signature genes linked to ESR include ABAT, CD24, GJA, KRT13, MSMB, PCP4 TF and THBS, among which CD24 and THBS were responsive to hypoxia, and CD24, GJA, KRT13 were involved in development of immune system, neuron projection and ectoderm, respectively. SRF (c-fos serum response element-binding transcription factor), the transcription factor which regulates the activity of many immediate-early genes, such as c-fos, has been known to participate in numerous significant processes like cell cycle regulation, apoptosis, and cell differentiation. It is the downstream target of many pathways including the mitogen-activated protein kinase pathway (MAPK) and is implicated in the hepatoma progression [19]. Among the four signature genes linked to SRF, Gro2 is a chemokine and an oncoprotein, Desmin an intermediate filament involved in muscle contraction and cytoskeleton organization, DLK a transmembrane protein is implicated in development of numerous cell types such as adipocytes, skeletal and neural systems and MLC2 is a myosin component involved in development of heart and muscle. The receptor directly connected to the majority of these TFs was EGFR, a tyrosine kinase coupled receptor known to be tightly associated with several cancers when overexpressed [20-22].

### Detection of developmental stages in cultured cells and embryonic tissue

As the network analysis above suggests possible roles of the 56 genes in tissue development and tumorigenesis, we intended to test whether the degree of similarity to our GETs may also reflect the physiological or developmental states of a given tissue. To proceed, we were able to obtain datasets for two embryonic tissues, skin and lung, from GEO.

**Figure 4:**
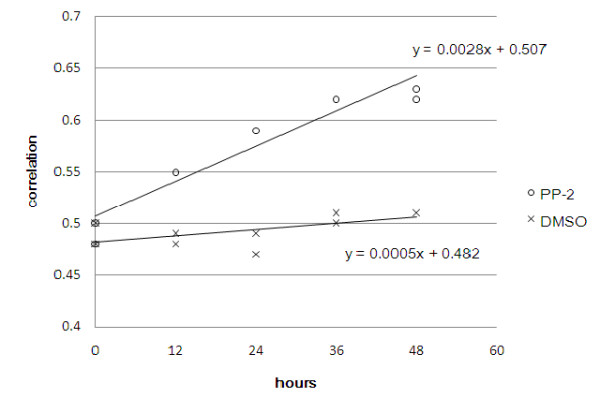
**The 56-gene profiles correlate with developmental progress of keratinocytes**. Correlation of the 56 genes was computed and plotted between the normal human skin and each sample from the dataset GSE6932 where NHEK cells were treated with PP-2 (open circle) or DMSO (cross) for various durations. The regression lines as well as the associated formula were also displayed with the plot. The slope of the regression line for the PP-2 treated NHEK is 0.0028 with p-value = 2 × 10^-5 ^on t-test, compared with the DMSO control whose regression line has slope of 0.0005 with p-value = 0.0531.

First we calculated the c.f. between our skin GET and the 56 gene expressions from each array of the dataset GSE6932 [23] where NHEK epidermal progenitor cells had been treated with 2-(3,4,5-trimethoxyphenylamino)-pyrrolo[2,3-d]pyrimidine (PP-2) for different periods of time. PP-2 can induce terminal differentiation of epidermal progenitor cells to keratinocytes. We found that the degree of similarity to adult skin as measured by correlation did increase with the duration of PP-2 induction (Figure 4): the slope of the regression line was 0.0028 with *P *value of t-test being 2 × 10^-5^.

**Figure 5:**
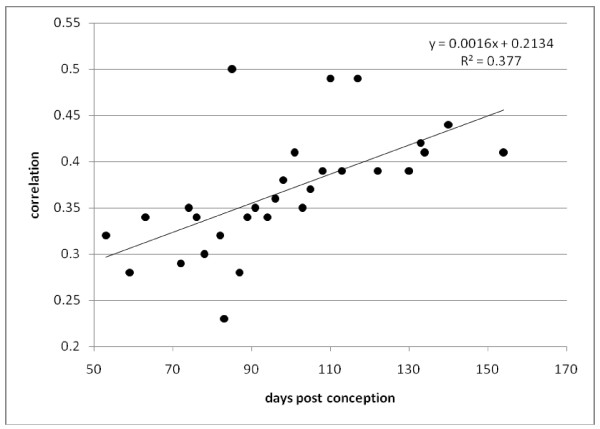
**The GET from adult lung in our training set positively correlates with development of human embryonic lungs**. Correlation between the 56-gene profiles from each of the human fetal lung samples (GSE14334) and that from our adult lung template were computed and plotted. The x-axis indicates age (in "days post conception") of the fetal samples and y-axis the correlation coefficient. As each of the samples in the test data came from different individuals, they may show genetic variation as well as different pathology. The slope of the regression line as displayed to the upper right corner was 0.0016 with P value = 0.00034 on t-test. The regression slopes for correlation with each of the 24 tissues was computed, and was highest with the lung samples. (see Additional file 1: Table S4).

The other dataset, GSE14334 [24], contained transcriptomic data derived from human embryonic lungs of ages ranging from 53 to 140 days post conception. We calculated the c.f.s of our adult lung GET and the 56-gene profiles of these fetal lungs. Again, we found an increasing trend with developmental stages (slope = 0.0016 with *P *values = 3 × 10^-4 ^on t-test, see Figure 5). This suggests that during the developmental process, gene expression in the embryonic cells gradually progresses toward the terminal equilibrium represented by the 56-gene profiles in the target adult tissues. The fluctuation observed in the correlation across developmental times may be caused by genetic variation or different pathological states among the donors.

To confirm that this trend was specific to the lung template only, not other tissue templates, we also performed the same correlation computation with each of the remaining 23 training tissues and calculated the regression coefficients of all correlation plots (Additional file 1: Table S7). The result agreed with our main finding that lung gave the highest regression coefficient among the twenty-four tissues.

### Deviation of normal expression profiles from cancerous tissue

Relationship of our normal-tissue derived GETs to gene expression of the cancerous tissues was firstly studied on the dataset GSE5364 which contained transcriptomic data from cancerous tissues. Correct tissue prediction was between 33%-97% (Table 2) and the c.f. to their corresponding tissues 0.4~0.73, both significantly lower than their paired normal parts. The decrease of prediction accuracy on cancerous tissues (Table 2) might reflect the increasingly heterogeneous nature of cancerous cells in a specimen. We applied the same tissue-prediction analysis to the transcriptomic data GSE5720 [25] from NCI60 cancer cell lines with a homogeneous progressing cell population. As expected, this analysis led to relatively poor prediction (Table 3), suggesting that the overall biochemical reactions in these cancerous cells may have become very different from those in a normal tissue. However, the significantly lower prediction accuracy using cell lines rather than cancerous tissues (Table 2 and Table 3) could be due to the intrinsic differences between these two systems.

**Table 2:** Tissue prediction on GSE5364 using as template the 56-gene profiles constructed from the 24 normal tissue types

Tissue/Cell line	Prediction Accuracy	Correlation Coefficient
Normal		
Liver^a ^(*n *= 8)	8 (100%)	0.85 ± 0.03
Lung^a ^(*n *= 12)	12 (100%)	0.81 ± 0.03
Thyroid^a ^(*n *= 16)	16 (100%)	0.81 ± 0.03
Cancer		
Hepatoma^b ^(*n *= 9)	3 (33%)	0.40 ± 0.16
Lung cancer^b ^(*n *= 18)	4 (22%)	0.61 ± 0.13
Thyroid cancer^b ^(*n *= 35)	34 (97%)	0.73 ± 0.06

^a ^the adjacent matched normal tissues of a primary tumor surgically removed from a cancer patient, according to the description of GSE5364 at GEO and the article by Yu et al. 2008.

^b ^a primary tumor sample surgically removed from a cancer patient, according to GSE5364 at GEO and the article by Yu et al. 2008.

**Table 3:** The 56-gene profiles in cancerous cells/tissues strongly deviate from that in the normal counterpart

NCI60 cell lines		
Tissue/Cell line	Prediction Accuracy^a^	Correlation Coefficient^b^
Lung (*n *= 8)	3 (37.5%)	0.21 ± 0.12
Ovary (*n *= 8)	0(0%)	0.15 ± 0.08
Prostate (*n *= 2)	0(0%)	0.05 ± 0.04
Kidney (*n *= 8)	7(87.5%)	0.43 ± 0.15
Skin ((*n *= 10)	0 (0%)	0.017 ± 0.05
**Skin-GSE3189**		
Normal skin (*n *= 7)	7 (100%)	0.78 ± 0.05
Benign nevus (*n *= 18)	17 (94.4%)	0.73 ± 0.06
Melanoma (*n *= 45)	5 (11.11%)	0.36 ± 0.14

^a ^Tissue prediction was as described in Methods by comparing the c.f. between the 56-gene profiles of the test against each of our 24 training tissues.

^b ^The c.f. exhibited here is that between the 56-gene profiles of the test and the corresponding normal tissue in our training set.

To further test whether our GETs would be capable of differentiating benign from malignant tumors, we analyzed dataset GSE3189 [26], from a melanoma study that had been extensively examined by various techniques. Melanoma is the most aggressive form of skin cancer, and its diagnosis is challenging even for seasoned pathologists. We found, in terms of both precision of tissue prediction and of correlation with our skin GET that the expression profiles of the 56-gene sets in most (40/45) of the malignant tumors strongly deviated from those in either normal skin or benign nevus. In short, we obtained 96% sensitivity and 89% specificity in distinguishing melanoma from normal skin tissue or benign skin tumor using the 56-gene profiles (Table 4). This result further supports our hypothesis that the combinatorial expression profile of the 56 genes accurately reflects the physiological state of a tissue. It also suggests that these 56-gene profiles could be used to facilitate diagnosis and as a sensitive research tool in cancer.

**Table 4:** Specificity and sensitivity of distinction of melanoma from nevi and normal skin using the 56-gene profile template.

	Normal or Benign	Melanoma	
Predict-to-skin	TP: 24	FP: 5	Positive predict: 29

Predict-to-other	FN: 1	TN: 40	Negative predict: 41

	Sensitivity = 96%	Specificity = 89%	

TP: true positives; FP: false positives; FN: false negatives; TN: true negatives

specificity = number of true negatives/[number of true negatives + number of false positives]

sensitivity = number of true positives/[number of true positives + number of false negatives]

### Distinguishing normal skin from skin substitutes

There is a potential application of our system in quality assessment in tissue engineering. To illustrate the concept, whether our 24 GETs can also characterize the normal state of the corresponding tissues. To answer the question, we used the dataset GSE3204 [27], which was originally produced to investigate the molecular differences between normal human skin (NHS) and cultured skin substitutes (CSS) in order to improve CSS for skin autograft in patients with massive skin loss caused by burn injury. The dataset investigated the whole-genome transcriptomes under four conditions: those in normal human skin (NHS), in cultured skin substitutes (CSS), in fibroblasts (CF) and keratinocytes (CK). CF and CK were used to prepare CSS. We computed the correlation of the 56-gene expression profiles between our skin GET and each sample in GSE3204, the NHS showed the highest correlation, followed by the CSS. CF and CK, the two constituent cells of CSS, were significantly lower than either of NHS and CSS (Table 5). This is a good reflection of the actual degree of similarity to normal skin.

**Table 5:** Distinction of native human skin from skin substitutes by the skin GET

Tissue/cell type	Correlation	Best matched tissue type
NHS (native human skin)	0.88 ± 0.03	Skin, skin, skin, skin

CSS (cultured skin substitutes)	0.74 ± 0.04	Skin, skin, skin, skin

CK (cultured keratinocytes)	0.61 ± 0.02	Skin, skin, skin, skin

CF (cultured fibroblasts)	0.35 ± 0.02	Uterus, lung, lung, lung

Data source: GSE3204

## Discussion

We have attempted to address the fundamental issue of "Can the normal physiological states of various human tissue types be quantified at the molecular level faithfully and succinctly?" In the biomedical literature, the phrase "normal physiological state" is often brought up to contrast the phrase "pathological or disease state". In physics or engineering, a "state" of a system must be quantified by well-defined variables. Can we do the same in the biological world? We conceptualized the issue by arguing that one way to describe a biological state at the molecular level is to present a template consisting of (a) a list of molecule species and (b) their relative abundance levels. To be useful, three properties should be possessed- compactness, repeatability and discrimination ability. The list should be reasonably short and the template should be able to predict the state accurately for as many sets of data generated by as many different labs as possible. Taking full advantage of the rich data resource provided by GEO (Gene Expression Omnibus), here we offered the characterization of normal physiological state a bench mark solution.

This report is the first to present a multi-purposed, molecule-based molecular model that can characterize as many as 24 different human tissue types. The success of our tissue-specific GETs in accurately predicting the tissue types from various sources and in discriminating tissues/cells at different developmental stages indicates that (A) a tissue under the disease-free condition constantly maintains certain stoichiometry among many gene products; (B) the same tissue type from different disease-free individuals shares very similar gene-product stoichiometry; (C) the gene-product stoichiometry can be expressed as the relative transcription levels of a set of representative genes, a gene expression template (GET) (the combinatorial expression levels of the 56 genes in this study); (D) When the physiological or developmental state of a cell shifts, the gene-product stoichiometry may change accordingly. (E)

Severe alteration from the normal state gene-product stoichiometry, possibly caused by multiple mutations in genes or dramatic shifts of the overall biochemical environment of a cell, may lead to abnormal growth like cancer, if not death of a cell. In support of this notion, we also demonstrated that the 56-gene expression patterns in cancerous cells/tissues significantly deviate from normal GET and that our tissue-specific GETs can be used to discern melanoma from benign nevi and from normal skin. Potential applications of our results to tissue engineering, cancer diagnosis and development studies are therefore inferred.

Our approach to constructing a gene signature for predicting tissue types is simpler than existing classification methods [28]. We first identified those genes showing a similar and reproducible trend in all three large datasets, then used the full gene group to perform tissue classification, and finally applied the group behavior (that is, the expression profiles of the compact 56-gene group) as predictors to characterize tissue types under various conditions. Without complicated modeling, our 56-gene signature provides high prediction power on numerous public datasets. As far as we are aware of, this is the most compact gene set capable of classifying the largest number of tissues. The use of multiple datasets which served as biological replicates allowed us to reduce the number of false positives and to find the genes with most variable expression across various tissues with better confidence. Note, however, that because of the high accuracy already achieved by the 56 genes, we did not explore the issue of possible existence of other gene sets that could serve as GETs and accomplish the same or even better rate of prediction - perhaps with aids of additional statistical tools such as one-way ANOVA for gene selection.

With the abundance of interplaying gene and pathway activities in a tissue, one may ask how the group behavior of these 56 genes can represent the states of various human tissue types. Our functional study of the 56 genes reveals a variety of functional categories including cytoskeleton (desmin, nebulin), signal transduction proteins (protein kinase C beta1, CDC28 protein kinase regulatory subunit 2), neural transmitter regulator (4-aminobutyrate aminotransferase), energy homeostasis regulator (insulin-like growth factor binding protein 1), and immunity (CD24 molecule) etc. It should be emphasized, however, that the high precision of large-scale validation on tissue prediction was not achieved through the combinatorial on/off states of a collection of tissue-specific markers because only 4 of the 56 genes appeared as tissue-specific genes which highly express in one particular tissue but minimally in others. They are *TFPI2 *specific for placenta, *ANKRD7 *for testis, *ELA2A *for pancreas and *APOC3 *for liver. However, the expressions of all 56 genes together as a template, did present distinctive patterns varying from tissue to tissue. Therefore, this gene set may be considered as the representative genes of the key biochemical pathways functioning distinctively across tissues, and the combinatorial transcription levels of the 56 genes, the GETs, may reflect the net sum of the relative activities of these pathways.

Despite that the feature of tissue-characterization of the 56 genes may not be exerted through collection of the so-called "tissue-specific" genes as discussed above, it would be interesting to find out how each of the 56 genes may contribute to tissue characterization. One of our on-going projects in reducing the gene set without compromising its power in defining the normal physiological state of a specified human tissue may help to answer this question.

The network analysis provided additional clues to the biological implications of the signature in development and carcinogenesis. Positive correlation of the 56- gene profile to developmental stages revealed in both in vitro and in vivo studies indicates that systematic shifts of the global gene expression through the complicated developmental process can be characterized with our signature genes. Hence it is possible that the 56 genes may be good candidates for modeling the human developmental process. Further, the capability of the 56- gene profiles in correlating quality of the engineered skin to the similarities to normal skin template brought up a potential application of the signature to serve as the quality index for engineered tissue.

The network analysis also helped to link our model to the current understanding of tumorigenesis. We showed that the c.f.s of the 56-gene profiles in malignant tumors were significantly lower than the normal tissues to the corresponding template, indicating changes of expression in multiple genes in a cancer tissue. It coincides with the findings that at least 4-5 mutations are required to initiate tumor [29,30]. In our network analysis, more than half of the 56 genes were found to interact with those well-known cancer-related transcription factors or signaling receptors such as STAT3, TP53, ESR1 and EGFR which have been shown to interact with a great number of gene products involved in varieties of pathways. Therefore, it is possible that mutations occurring in such genes (i.e. *EGFR, STAT3 *etc.) may simultaneously affect expression of a number of the target genes which may ultimately lead to changes in the profile of our signature. Further, significant change in the profile of the 56 genes indicates alterations in relative activities of the pathways represented by these signature genes, reflecting a dramatic shift of the cellular homeostasis which may lead to cell necrosis or anomalous growth like tumorigenesis. Alternatively, accumulated mutations in the genes which affect the activities of those pathways represented by our signature may also affect the expression profile in one hand and lead to similar outcome as described above on the other hand. Taken together, despite that severe shift of the 56-gene profile from normal may not be the initial cause of many cancers, it could have the potential to serve as an indicator for the cancerous state of a cell/tissue. Whether our signature can be applied to cancer staging awaits further investigation. Nonetheless, this knowledge provides a new aspect in understanding the complex process of carcinogenesis.

## Conclusions

These results strongly suggest that a normal tissue or cell may uphold its normal morphology and functioning by maintaining specific chemical stoichiometry among genes. The stoichiometry of a physiological state of a normal human tissue can be depicted by the relative expression levels of a compact set of representative genes such as the 56 genes obtained here. A significant deviation from such quantitative relationship may result in malfunction or abnormal growth of the cells.

## Methods

### Data

Microarray data used in this study were obtained from the Gene Expression Omnibus (GEO) database at NCBI by Nov. 2^nd ^of 2009. GEO series with accession numbers GSE2361[4], GSE1133[6](2004 version of the Gene Atlas) and GSE7307[31] (the "human body index") were used to find molecular features in normal tissues and to derive the 56-gene template profiles. (Additional file 1: Table S1) Datasets GSE14334, GSE3204, GSE5364 and GSE6932 were used as testing data to further explore the biological implications of GETs. Datasets GSE1133, GSE2361, GSE5364 and GSE6932 were hybridized on the Affymetrix GeneChip HG-U133A and GSE7307 on the HG-U133plus2.0. The Affymetrix GeneChip HG-U133plus2.0 contained 54,675 probe sets (representing around 38,572 unique UniGene clusters) which cover all the 22283 probe sets (representing 14,593 unique UniGene clusters) synthesized on the HG-U133A. The additional 62 datasets used for large-scale tissue prediction had all been hybridized on either HG-U133A or HG-U133plus2.0. The accession identification as well as the associated information are summarized in Additional file 1: Tables S1 and S3.

### Molecular annotation for selected genes

The gene sets were annotated by searching the databases at the DAVID server (http://david.abcc.ncifcrf.gov/home.jsp) with Entrez Gene [32] identifier as input. Cellular location and biological processes were searched against Gene Ontology (GO) [33]. The molecular functions were searched against PANTHER[34], since PANTHER gave a more complete set of biologically-relevant results for our gene set than GO. Pathways were searched against KEGG [35].

### Microarray Analysis

For those datasets whose CEL files are available at GEO, the data were first subjected to quality assessment by AffyQualityReport to remove the poor quality arrays and then to RMA[36] processing for data normalization.

For identification of the 56 signature genes, this preprocessing procedure resulted in 143, 35 and 473 arrays for GSE1133, GSE2361, and GSE7307, respectively. Gene filtration was carried out by firstly selecting from each of the three training datasets the genes whose coefficients of variation ranked at top 2.5% of the entire transcriptome across different tissue types. The resulted highly variably expressed genes were then intersected to generate a set of candidate tissue-classifier genes which were later subjected to data redundancy elimination through hierarchical clustering against the 24 tissues commonly present in the three sets of training data. Following the hierarchical cluster analysis, one representative gene for each cluster was selected and additional genes with highly similar expression profiles got removed. This procedure resulted in 56 genes.

For tissue classification, the probe set intensities of the 56 genes or an equivalent number of random probe sets of the 24 selected tissues were extracted from each of the three GEO datasets using the programs Microsoft Access and Excel. The extracted probe intensities from the three datasets were then combined into a 56 × 72 matrix which was then subjected to hierarchical clustering with the GenePattern package [37] using Pearson correlation for similarity computing and average for clustering. Ten sets of 56 random probe sets were produced by a random number generation program written in C. Each set was used for a separate hierarchical clustering analysis.

Both AffyQualityReport and RMA were obtained from the Bioconductor package [38] in the R package (http://www.r-project.org/). Descriptive statistical analyses were computed using Excel while hierarchical clustering with the GenePattern package.

### Tissue prediction using the 56 genes

Tissue prediction was performed following the KNN method (k-nearest neighbor) with k = 1. It compares the c.f. of the 56-gene profiles between a test tissue and each of our 24 tissue-specific GET profiles, one for each tissue type. The tissue type with highest correlation was nominated as our prediction. A computer program in R language was implemented to accomplish this task.

### Dataset retrieval from GEO for large-scale tissue-prediction

Text The entire GEO database (2009-11-2 freeze) was searched with the following criteria: platform as GPL96 (Affymetrix HG-U133A) or GPL570 (HG-U133plus2.0), sample source containing one of the 24 distinguishable human organ/tissues and key word in the sample-related fields containing "normal". Two bioinformatics strategies were used to carry out the search: one was to apply SQL commands to the local MySQL database housing the data from the soft files of GPL96 and GPL570 which were imported from GEO website. The other strategy was to directly query the GEO database with Entrez keywords through the NCBI web interface. The union of both searching results was taken, followed by manual filtration to exclude irrelevant datasets that, for example, came from cell lines or specific cell types. Those datasets which had been contributed by the same research group as the three source datasets, GSE3526 for instance, were also removed from our test set. Expression profiles of the 56 genes were then extracted from the 61 resulting datasets.

Datasets of 56 gene expression values were organized into RMA-like or MAS-like according to the data preprocessing methods. For those datasets that had been normalized with MAS5 or equivalent method, logarithmic transformation was carried out prior to tissue-prediction analysis. For three datasets (GSE13355, GSE14951, GSE17539) it was hard to judge whether logarithm transformation was necessary and their CEL files were therefore preprocessed with AffyQualityReport followed by RMA normalization before tissue-prediction analysis.

### Gene network construction

Gene networks were constructed with the MetaCore package using the algorithms "network analysis" and "receptor targets modeling". The algorithms are variants of the shortest paths algorithm where the main parameters are: 1) relative enrichment with the uploaded data (the 56 genes in this study), and 2) relative saturation of networks with canonical pathways. As a control for this network analysis, a set of 56 genes randomly selected from the Affymetrix microarray HG-U133A was entered as a query and no network was produced by either of the algorithms. The control experiments were repeated twice.

## List of Abbreviations

GET: tissue-specific gene expression templates.

## Authors' contributions

PH conceived of the study, participated in its design and coordination, performed data analysis, interpreted the results and drafted the manuscript. HW participated in preprocessing and statistical computation of the training data. CW helped to build the procedure to obtain the signature genes and implemented computer programs for tissue prediction. CC carried out the batch search/retrieval of the test data from GEO. BL participated in intellectual discussion regarding biological implication of GET. SY and GW helped to maintain the computation environment and participated in statistical discussion. KL participated in intellectual discussion, helped to revise the manuscript and supervised the research team. All authors read and approved the final manuscript.

## Supplementary Material

Additional file 1 **Table S1. Summary of three datasets**. Table S2. The over-represented KEGG pathways mapped with the 56 genes. Table S3. Tissue prediction on the normal human tissues in GSE5364 using as template the 56-gene profiles constructed from the 24 normal tissue types. Table S4. Results of the large-scale tissue prediction with the 56-gene templates organized by dataset. Table S5. Large-scale prediction of normal human tissues with GETs using Spearman correlation. Table S6. Tissue prediction on the ABI-platform based dataset GSE 7905 using the 56-gene template. Table S7. Regression slopes of the correlations between the 56-gene profiles from fetal lungs and that from each of the 24 tissue GETs. Figure S1- Hierarchical clustering analysis on the 24 tissues by the 56 signature genes. Figure S2- Heat map of hierarchical clustering of the randomly selected 56 probe sets. Figure S3- Dendrogram of the hierarchical clustering results for tissue classification by the most expressed genes. Click here for file
