# Supplementary material for: Tissue-specific gene expression templates for accurate molecular characterization of the normal physiological states of multiple human tissues with implication in development and cancer studies
Source: BMC Genomics. 2011 Sep 1;12:439. doi: 10.1186/1471-2164-12-439 (PMC3178546; doi:10.1186/1471-2164-12-439)
Supplement: Additional file 1 — Table S1. Summary of three datasets. Table S2. The over-represented KEGG pathways mapped with the 56 genes. Table S3. Tissue prediction on the normal human tissues in GSE5364 using as template the 56-gene profiles constructed from the 24 normal tissue types. Table S4. Results of the large-scale tissue prediction with the 56-gene templates organized by dataset. Table S5. Large-scale prediction of normal human tissues with GETs using Spearman correlation. Table S6. Tissue prediction on the ABI-platform based dataset GSE 7905 using the 56-gene template. Table S7. Regression slopes of the correlations between the 56-gene profiles from fetal lungs and that from each of the 24 tissue GETs. Figure S1- Hierarchical clustering analysis on the 24 tissues by the 56 signature genes. Figure S2- Heat map of hierarchical clustering of the randomly selected 56 probe sets. Figure S3- Dendrogram of the hierarchical clustering results for tissue classification by the most expressed genes. [file 1471-2164-12-439-S1.PDF]

Additional File 1

Tissue-specific gene expression templates for accurate molecular characterization of the normal physiological states of multiple human tissues with implication in development and cancer studies

Pei-Ing Hwang[1], Huan-Bin Wu[1], Chin-Di Wang[1], Bai-Ling Lin[2], Cheng-Tao Chen[1], Shinsheng Yuan[1], Guani Wu[1], and Ker-Chau Li[1,2,3 §]

[1]Institute of Statistical Science, Academia Sinica, Taipei, Taiwan 115, Republic of China

[2]Genome Research Center, Academia Sinica, Taipei, Taiwan 115, Republic of China

[3]Department of Statistics, University of California, Los Angeles, Los Angeles, California 90095, USA

[§]Corresponding author

Supplementary Tables and Figures

Table of Content

(1) Table S1. Summary of three datasets

(2) Table S2. The over-represented KEGG pathways mapped with the 56 genes

(3) Table S3. Tissue prediction on the normal human tissues in GSE5364 using as template the 56-gene profiles constructed from the 24 normal tissue types

(4) Table S4. Results of the large-scale tissue prediction with the 56-gene templates organized by dataset

(5) Table S5. Large-scale prediction of normal human tissues with GETs using Spearman correlation

(6) Table S6. Tissue prediction on the ABI-platform based dataset GSE 7905 using the 56 gene template

(7) Table S7. Regression slopes of the correlations between the 56-gene profiles from fetal lungs and that from each of the 24 tissue GETs.

(8) Figure S1– Hierarchical clustering analysis on the 24 tissues by the 56 signature genes.

(9) Figure S2– Heat map of hierarchical clustering of the randomly selected 56 probe sets

(10) Figure S3– Dendrogram of the hierarchical clustering results for tissue classification by the most expressed genes

Table S1. Summary of the three GEO data series used in this study

Data Set	Platform	Chips	Normal Tissues	Replicates/ Tissue	Publication
**GSE 7307 (human body index)**	Affymetrix HG-U133 plus 2.0	473	86	1 ~ 21 (median = 3; mean = 4)	FASEB J 2005 (On RNA degradation)
**GSE 2361**	Affymetrix HG-U133A	35	35	1	Genomics 2005 (Comparison with cancer)
**GSE 1133 (GNF_2004)**	Affymetrix HG-U133A	143	66	1 ~ 2	PNAS 2004 (Human and mouse transcriptome)

Table S2. The over-represented KEGG pathways mapped with the 56 genes

Term	Count	%	PValue	Genes	List Total	Pop Hits
hsa04510:Focal adhesion	6	10.7	8.76E-04	209875_S_AT, 207957_S_AT, 209742_S_AT, 201110_S_AT, 209156_S_AT, 201058_S_AT	21	201
hsa04512:ECM-receptor interaction	4	7.1	0.004	209875_S_AT, 206488_S_AT, 201110_S_AT, 209156_S_AT	21	84

hsa03320:PPAR signaling pathway	3	5.4	0.029	206488_S_AT, 203980_AT, 205820_S_AT	21	69

Table S3. Tissue prediction on the normal human tissues in GSE5364 using as template the 56-gene profiles constructed from the 24 normal tissue types

**Tissue / Cell line**	**Prediction Accuracy**
**Normal**	
**Liver (*n* = 8)**	8 (100%)
**Lung(*n* = 12)**	12 (100%)
**Thyroid(*n* = 16)**	16 (100%)

Table S4. Results of the large-scale tissue prediction with the 56-gene templates organized by dataset

data set	tissue	# normal samples	#correct_ prediction	% accuracy	reference
GSE16334	bone marrow	11	11	100%	Blood. 2009, 114(26):5290-8
GSE15238	fetal liver	6	6	100%	PLoS One (2009), 4(10):e7511.
GSE1145	heart	11	11	100%	Not provided
GSE2719	heart	1	1	100%	J Surg Res (2006), 135(2):282-90.
GSE5406	heart	16	16	100%	Circulation (2006), 114(12):1269-76.
GSE11151	kidney	3	3	100%	Int J Biol Sci (2009) , 5(6):517-27.
GSE12606	kidney	4	4	100%	Cancer Immunol Immunother (2009), 58(9):1407-17.
GSE15641	kidney	23	23	100%	Clin Cancer Res(2005),

					11(16):5730-9.
GSE17861	kidney	16	16	100%	Transpl Int (2009), 22(3):293-302.
GSE2004	kidney	7	7	100%	Not provided
GSE2719	kidney	1	1	100%	J Surg Res (2006), 135(2):282-90.
GSE6280	kidney	2	2	100%	Oncogene (2008), 27: 5578–5589
GSE7392	kidney	22	21	95%	BMC Genomics(2007), 8:88.
GSE781	kidney	5	5	100%	BMC Cancer (2003), 3:31
GSE8050	kidney	2	2	100%	Cancer Res (2008), 68(7):2447-54.
GSE9493	kidney	23	22	96%	Am J Transplant(2009), 517-26.
GSE12720	liver	33	33	100%	Am J Transplant(2009), 9(4):758-72.
GSE13471	liver	5	5	100%	Nat Genet (2009) , 41(2):178-86.
GSE14951	liver	13	13	100%	Liver Transpl (2007), 13(1):99-113.
GSE2004	liver	6	6	100%	Not provided
GSE2719	liver	1	1	100%	J Surg Res (2006), 135(2):282-90.
GSE5364	liver	8	8	100%	PLoS Genet (2008), 4(7):e1000129.
GSE6222	liver	2	2	100%	*Oncogene* (2008), 27:5578–5589
GSE6764	liver	10	10	100%	Hepatology (2007), 45(4):938-47.
GSE15239	liver	6	6	100%	Not provided
GSE10072	lung	49	49	100%	PLoS One(2008), 3(2):e1651.
GSE10799	lung	3	3	100%	Clin Cancer Res (2009), 15(5):1566-74.
GSE15240	lung	1	1	100%	Cancer Res (2009), 69(8):3364-73.

GSE1643	lung	40	40	100%	Am J Respir Cell Mol Biol (2006), 35(1):65-71.
GSE16538	lung	6	6	100%	Am J Respir Crit Care Med (2009), 179(10):929-38.
GSE2719	lung	1	1	100%	J Surg Res (2006), 135(2):282-90.
GSE3268	lung	5	5	100%	Bioinformatics (2005), 21(23):4205-8
GSE5364	lung	12	12	100%	PLoS Genet (2008), 4(7):e1000129.
GSE7670	lung	30	30	100%	BMC Genomics (2007) , 8:140.
GSE8581	lung	19	18	95%	Am J Respir Cell Mol Biol (2009), 40(3):359-67.
GSE2549	lung	4	4	100%	American Journal of Pathology(2005), 166: 1827-1840.
GSE6008	ovary	4	4	100%	J Biol Chem (2010),285(3):1928-38.
GSE16515	pancreas	16	13	81%	Cancer Cell (2009), 16(3):259-66.
GSE2719	pancreas	1	1	100%	J Surg Res (2006) , 135(2):282-90.
GSE2175	pituitary gland	1	1	100%	Eur J Endocrinol (2005), 153(1):143-51.
GSE12767	placenta	8	8	100%	Placenta (2009), 30(1):15-24.
GSE13155	placenta	2	2	100%	Mol Syst Biol (2009), 5:279.
GSE14722	placenta	23	23	100%	Endocrinology (2009), 150(1):452-62.
GSE6573	placenta	1	1	100%	Hypertension (2007) , 49(3):604-11.
GSE17906	prostate	5	5	100%	BMC Cancer (2009), 9:317.

GSE2719	prostate	1	1	100%	J Surg Res (2006), 135(2):282-90.
GSE8218	prostate	4	4	100%	Not provided
GSE2719	skeletal muscle	1	1	100%	J Surg Res (2006), 135(2):282-90.
GSE6011	skeletal muscle	14	14	100%	FASEB J (2007), 21(4):1210-26.
GSE11971	skeletal_muscle	4	4	100%	BMC Immunol (2008) Jul 31;9:43.
GSE1462	skeletal_muscle	3	3	100%	FASEB J (2005), 19(7):866-8.
GSE3307	skeletal_muscle	5	5	100%	Brain (2006), 129(Pt 4):996-1013
GSE5086	skeletal_muscle	81	81	100%	PLoS Genet (2006), 2(7):e115
GSE6798	skeletal_muscle	13	13	100%	Diabetes (2007), 56(9):2349-55.
GSE8157	skeletal_muscle	13	13	100%	PLoS One (2008), 3(6):e2466.
GSE13355	skin	64	64	100%	Nat Genet (2009) , 41(2):199-204.
GSE14905	skin	21	21	100%	PLoS One (2008), 3(7):e2737.
GSE17539	skin	4	4	100%	J Invest Dermatol (2010), 130(2):587-601.
GSE2503	skin	6	6	100%	Mol Cancer (2006), 8;5:30.
GSE2719	skin	1	1	100%	J Surg Res (2006), 135(2):282-90.
GSE3189	skin	7	7	100%	Clin Cancer Res (2005), 11(20):7234-42.
GSE4587	skin	2	2	100%	Cancer Biol Ther (2005), 4(9):1018-29.
GSE5667	skin	5	5	100%	Exp Dermatol (2007), 16(1):28-36.
GSE6281	skin	7	7	100%	J Invest Dermatol

					(2007), 127(11):2585-95.
GSE7553	skin	4	4	100%	BMC Med Genomics (2008), 1:13.
GSE3218	testis	6	6	100%	Cancer Res (2006), 66(2):820-7.
GSE3467	thyroid	9	9	100%	Proc Natl Acad Sci USA (2005), 102(52):19075-80.
GSE3678	thyroid	7	7	100%	Not provided
GSE5364	thyroid	16	16	100%	PLoS Genet (2008), 4(7):e1000129.
GSE6004	thyroid	4	4	100%	Proc Natl Acad Sci USA (2007), 04(8):2803-8.
GSE13319	uterus	27	27	100%	Cancer Res (2009), 69(15):6171-8.
Total		797	791	99.2%	

Table S5. Large-scale prediction of normal human tissues with GETs using Spearman correlation

**Tissue**	**Datasets**	**Samples**	**Correctly predicted**	**% Accuracy**
bone marrow	1	11	11	100%
fetal liver	1	6	6	100%
heart	3	28	28	100%
kidney	11	108	103	95.4%
liver	9	84	79	94.1%
lung	11	170	170	100%

ovary	1	4	4	100%
pancrease	2	17	16	94.1%
pituitary gland	1	1	1	100%
placenta	4	34	34	100%
prostate	3	10	10	100%
skeletal muscle	8	134	124	92.5%
skin	10	121	121	100%
testis	1	6	6	100%
thyroid	4	36	36	100%
Uterus	1	27	18	66.67%
Total	61	797	767	96.23%

Table S6. Tissue prediction on the ABI-platform based dataset GSE 7905 using the 56 gene template

Accession	Sample Title	prediction
GSM194459	Human fetal liver replicate 1 of 3	fetal_liver
GSM194460	Human fetal liver replicate 2 of 3	fetal_liver
GSM194461	Human fetal liver replicate 3 of 3	fetal_liver
GSM194462	Human lung replicate 1 of 3	Lung
GSM194463	Human lung replicate 2 of 3	Lung

GSM194464	Human lung replicate 3 of 3	Lung
GSM194465	Human liver replicate 1 of 3	Liver
GSM194466	Human liver replicate 2 of 3	Liver
GSM194467	Human liver replicate 3 of 3	Liver
GSM194474	Human fetal brain replicate 1 of 3	fetal_brain
GSM194475	Human fetal brain replicate 2 of 3	fetal_brain
GSM194476	Human fetal brain replicate 3 of 3	fetal_brain
GSM194477	Human prostate replicate 1 of 3	Prostate
GSM194478	Human prostate replicate 2 of 3	Prostate
GSM194479	Human prostate replicate 3 of 3	Prostate
GSM194480	Human skeletal muscle replicate 1 of 3	skeletal_muscle
GSM194481	Human skeletal muscle replicate 2 of 3	skeletal_muscle
GSM194482	Human skeletal muscle replicate 3 of 3	skeletal_muscle
GSM194483	Human heart replicate 1 of 3	Heart
GSM194484	Human heart replicate 2 of 3	Heart
GSM194485	Human heart replicate 3 of 3	Heart
GSM194486	Human spinal cord replicate 1 of 3	spinal_cord
GSM194487	Human spinal cord replicate 2 of 3	spinal_cord
GSM194488	Human spinal cord replicate 3 of 3	spinal_cord
GSM194492	Human trachea replicate 1 of 3	trachea
GSM194493	Human trachea replicate 2 of 3	trachea
GSM194494	Human trachea replicate 3 of 3	trachea
GSM194495	Human uterus replicate 1 of 3	Uterus
GSM194496	Human uterus replicate 2 of 3	Uterus
GSM194497	Human uterus replicate 3 of 3	Uterus
GSM194501	Human skin replicate 1 of 3	skin
GSM194502	Human skin replicate 2 of 3	skin
GSM194503	Human skin replicate 3 of 3	skin
GSM194504	Human ovary replicate 1 of 3	Ovary
GSM194505	Human ovary replicate 2 of 3	Ovary
GSM194506	Human ovary replicate 3 of 3	Ovary
GSM194507	Human testis replicate 1 of 3	testis
GSM194508	Human testis replicate 2 of 3	testis
GSM194509	Human testis replicate 3 of 3	testis
GSM194510	Human pancreas replicate 1 of 3	Pancreas
GSM194511	Human pancreas replicate 2 of 3	Pancreas
GSM194512	Human pancreas replicate 3 of 3	Pancreas

GSM194513	Human thymus replicate 1 of 3	thymus
GSM194514	Human thymus replicate 2 of 3	thymus
GSM194515	Human thymus replicate 3 of 3	thymus
GSM194519	Human kidney replicate 1 of 3	kidney
GSM194520	Human kidney replicate 2 of 3	kidney
GSM194521	Human kidney replicate 3 of 3	kidney
GSM194525	Human placenta replicate 1 of 3	PLACENTA
GSM194526	Human placenta replicate 2 of 3	PLACENTA
GSM194527	Human placenta replicate 3 of 3	PLACENTA
GSM194528	Human thyroid replicate 1 of 3	Thyroid
GSM194529	Human thyroid replicate 2 of 3	Thyroid
GSM194530	Human thyroid replicate 3 of 3	Thyroid
GSM194531	Human salivary gland replicate 1 of 3	salivary_gland
GSM194532	Human salivary gland replicate 2 of 3	salivary_gland
GSM194533	Human salivary gland replicate 3 of 3	salivary_gland
GSM194549	Human bone marrow replicate 1 of 3	bone_marrow
GSM194550	Human bone marrow replicate 2 of 3	bone_marrow
GSM194551	Human bone marrow replicate 3 of 3	bone_marrow

Table S7. Regression slopes for plots of the correlation between the 56-gene profiles from the human embryonic lungs and that from each of the 24 tissue-specific GETs.

tissue	slope
Lung	0.00158
skin	0.001218
salivary_gland	0.001158
trachea	0.001103
thymus	0.000928
Liver	0.000437
Pancreas	0.00037
bone_marrow	0.000324
skeletal_muscle	0.000153
Thyroid	0.00012
Heart	3.09E-05
kidney	-9.45E-05
Prostate	-0.00026
Uterus	-0.00049
amygdala	-0.0006
PLACENTA	-0.00077
fetal_liver	-0.00084
spinal_cord	-0.00097
Ovary	-0.00103
thalamus	-0.00105
cerebellum	-0.0011
fetal_brain	-0.00128
testis	-0.00136
pituitary_gland	-0.00161

C.f. for the 56-gene profiles between the embryonic lung (GSE14334) and each of our 24 tissue-specific GETs was firstly calculated using the R software package. The table shows the slopes of the derived linear regression lines for each of the 24 GETs by taking the c.f. as Y and the age (in days post conception) as X using the R program "lm". Please note that the slope with human adult lung ranks at top. The scatter plot of Figure 4 in the main text illustrates that the correlation of the 56-gene of the adult lung increases with ages of embryonic lungs.

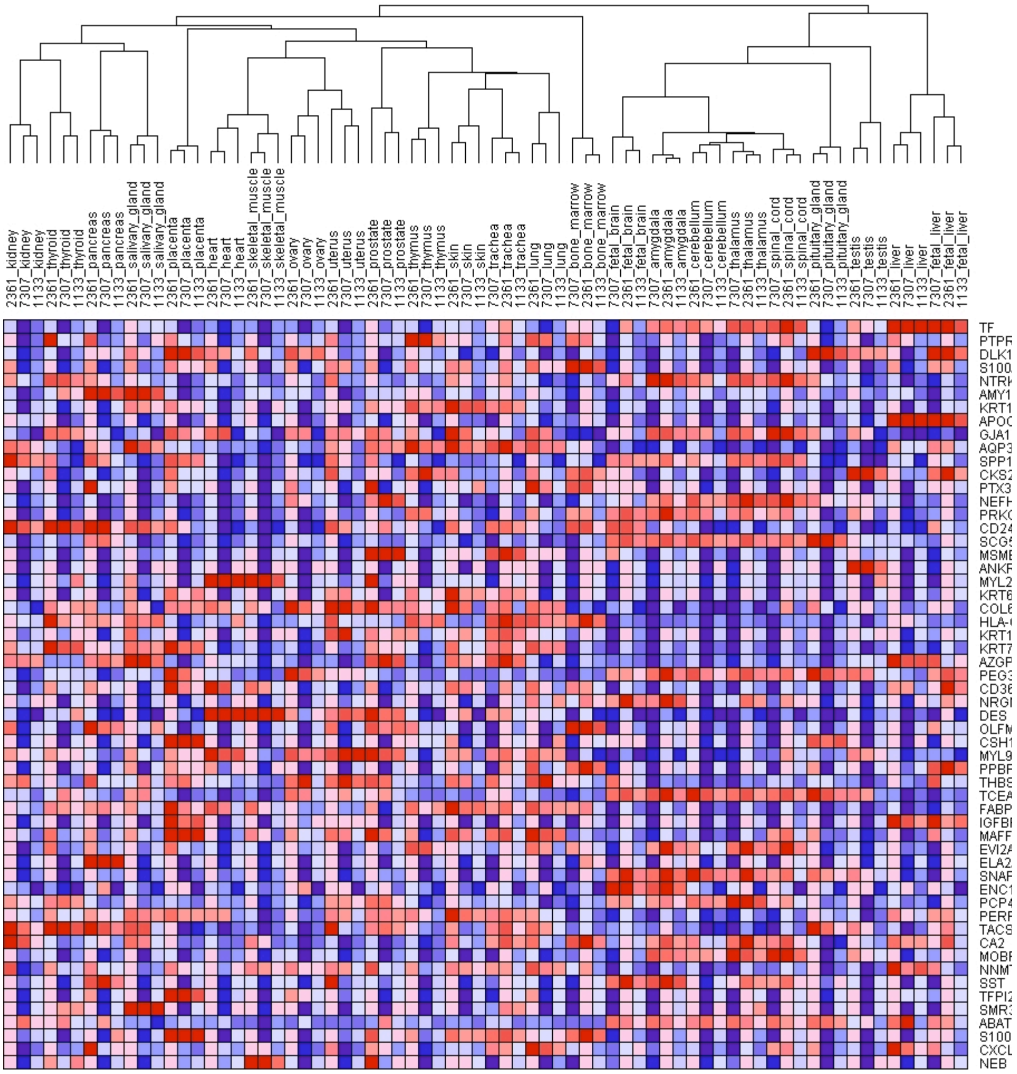

Figure S1. The 56 signature genes resulted in complete tissue classification using hierarchical clustering analysis.

Expression data of the 56 signature genes were extracted and subjected to standard one-way hierarchical clustering analysis against the common 24 tissues from the three GSE datasets. The columns indicate the tissue origins of the samples and the rows the signature genes whose gene symbols are displayed to the right of their representing rows. The dendrogram shown on top of the heat map indicates clustering of the 24 tissues.

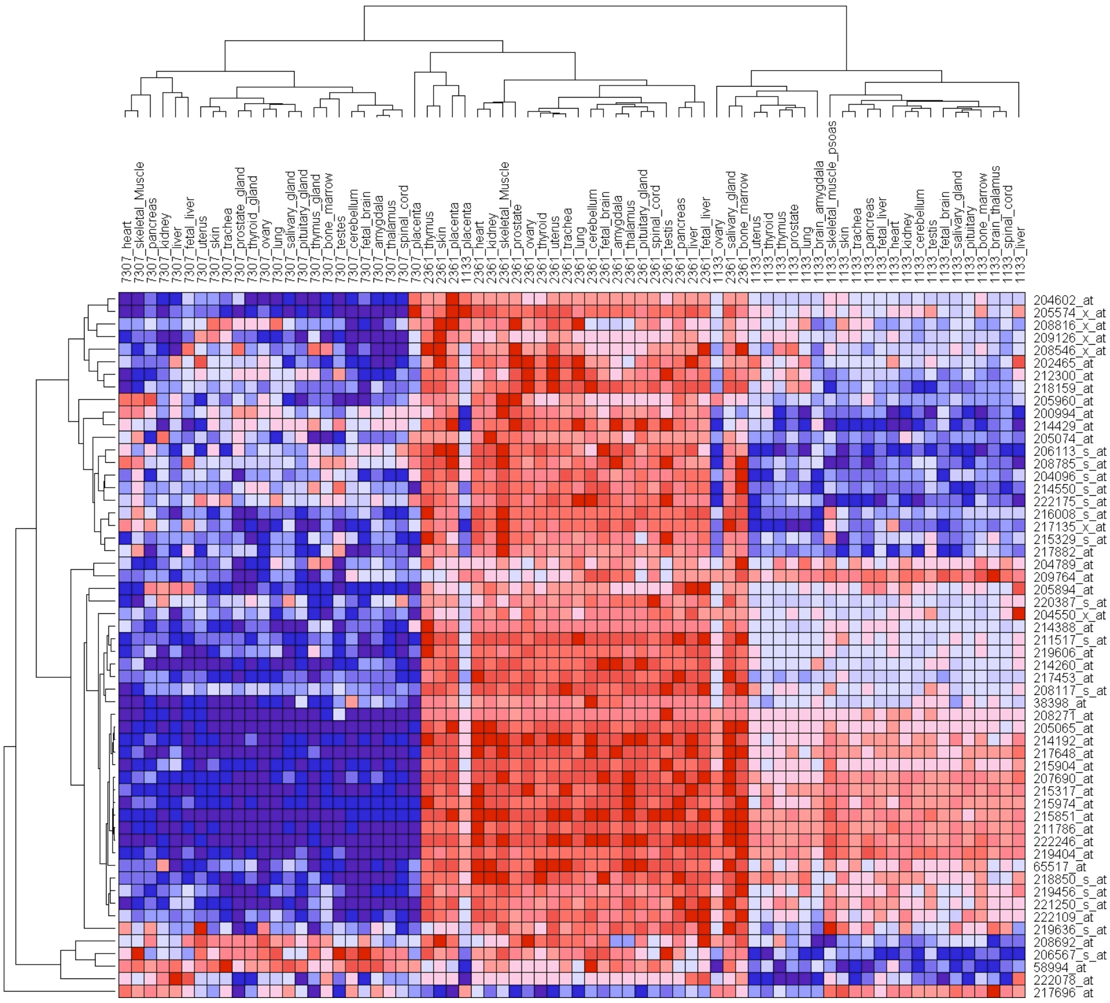

Figure S2. Heat map of hierarchical clustering analysis using 56 randomly selected genes against the 24 common tissues.

The columns of the heat map indicate the tissues which were annotated on the top of the map and the rows the 56 random probe sets annotated to the right. Please note that all 72 conditions (24 tissues times 3 datasets) were grouped into three large groups largely following their source datasets. Displayed here is one of the ten replicate analyses.

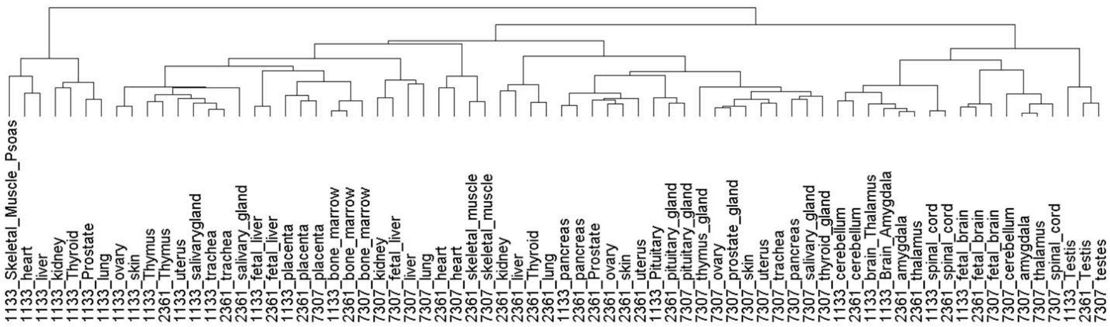

Figure S3 – Dendrogram of the hierarchical clustering results for tissue classification by the most expressed genes

Tissue classification effect was also tested on the most expressed genes. 448 transcripts which showed the highest expression in all the three training datasets were selected for hierarchical clustering analysis. The same procedure as in the Figure S2 was followed with probe sets replaced for these 448 abundant transcripts.
